# Cost trends of metastatic renal cell carcinoma therapy: the impact of oral anticancer agents and immunotherapy

**DOI:** 10.1093/jncics/pkae067

**Published:** 2024-08-12

**Authors:** Rebecca Forman, Jessica B Long, Sarah J Westvold, Khushi Agnish, Hannah D Mcmanus, Michael S Leapman, Michael E Hurwitz, Lisa P Spees, Stephanie B Wheeler, Cary P Gross, Michaela A Dinan

**Affiliations:** Section of Medical Oncology, Internal Medicine Department, Yale School of Medicine, New Haven, CT, USA; Yale Cancer Outcomes, Public Policy, and Effectiveness Research (COPPER) Center, New Haven, CT, USA; Department of Internal Medicine, Yale School of Medicine, New Haven, CT, USA; Yale Cancer Outcomes, Public Policy, and Effectiveness Research (COPPER) Center, New Haven, CT, USA; Department of Internal Medicine, Yale School of Medicine, New Haven, CT, USA; Yale School of Medicine, New Haven, CT, USA; Department of Medicine, Duke University School of Medicine, Durham, NC, USA; Yale Cancer Outcomes, Public Policy, and Effectiveness Research (COPPER) Center, New Haven, CT, USA; Department of Urology, Yale School of Medicine, New Haven, CT, USA; Section of Medical Oncology, Internal Medicine Department, Yale School of Medicine, New Haven, CT, USA; Department of Health Policy and Management, Gillings School of Global Public Health, University of North Carolina at Chapel Hill, Chapel Hill, NC, USA; Department of Health Policy and Management, Lineberger Comprehensive Cancer Center, University of North Carolina at Chapel Hill, Chapel Hill, NC, USA; Department of Health Policy and Management, Gillings School of Global Public Health, University of North Carolina at Chapel Hill, Chapel Hill, NC, USA; Department of Health Policy and Management, Lineberger Comprehensive Cancer Center, University of North Carolina at Chapel Hill, Chapel Hill, NC, USA; Yale Cancer Outcomes, Public Policy, and Effectiveness Research (COPPER) Center, New Haven, CT, USA; Department of Internal Medicine, Yale School of Medicine, New Haven, CT, USA; Yale Cancer Outcomes, Public Policy, and Effectiveness Research (COPPER) Center, New Haven, CT, USA; Department of Chronic Disease Epidemiology, Yale School of Public Health, New Haven, CT, USA

## Abstract

**Background:**

Immunotherapy (IO) and oral anticancer agents (OAA) have improved outcomes for metastatic renal cell carcinoma (mRCC), but there is a need to understand real-world costs from the perspective of payers and patients.

**Methods:**

We used retrospective fee-for-service Medicare 100% claims data to study patients diagnosed with mRCC in 2015-2019. We identified initial treatment type and costs (the year after diagnosis) and analyzed differences in monthly and 12-month costs over time and between OAA, IO, and combination groups and the association between Out-Of-Pocket (OOP) costs and adherence.

**Results:**

We identified 15 407 patients with mRCC (61% male; 85% non-Hispanic White). A total of 6196 received OAA, IO, or combination OAA/IO as initial treatment. OAA use decreased (from 31% to 11%) with a simultaneous rise in patients receiving IO (3% to 26%) or combination IO/OAA therapy (1% to 11%). Medicare payments for all patients with mRCC increased by 41%, from $60 320 (95% confidence interval = 58 260 to 62 380) in 2015 to $85 130 (95% confidence interval = 82 630 to 87 630) in 2019. Payments increased in patients who received OAA, IO, or combination OAA/IO but were stable in those with other/no treatment. Initial higher OOP responsibility ($200-$1000) was associated with 13% decrease in percent days covered in patients receiving OAA in the first 90 days of treatment, compared with those whose OOP responsibility was less than $200.

**Conclusion:**

From 2015 to 2019, costs for Medicare patients with mRCC rose substantially due to more patients receiving IO or IO/OAA combined therapy and increases in costs among those receiving those therapies. Increased OOP costs was associated with decreased adherence.

First-line treatment of metastatic renal cell carcinoma (mRCC) has dramatically changed since the 1990s, when cytokine therapy was standard of care, despite minimal survival improvements and significant toxicity (1). From 2003 to 2017, oral anticancer agents (OAAs) showed improved overall survival (OS) and were approved for treatment of mRCC (2-5). In 2015, immunotherapy (IO) was approved in the anti-angiogenic treatment refractory setting and was used off-label in the first-line setting. In 2018, ipilimumab and nivolumab were approved in the first-line setting based on improvements in objective response, with responses proving durable at the 4-year mark (6). Since 2018, the treatment paradigm has included dual-agent IO or combinations of IO and OAA agents (7). With increasingly effective therapies, OS improved from less than 1 year in the 1990s to more than 5 years currently with substantial decrease in treatment toxicities (8).

Although OAAs and IO provide substantial clinical benefits, they are expensive, with several in the 100 most expensive drugs paid for by Medicare (9). Adding a targeted therapy such as OAAs to a chemotherapy regimen can increase the price sixfold (10), and treatment costs have risen at a rate far greater than that of inflation or traditional chemotherapies (11). The impact of OAAs and IO on mRCC costs specifically, however, and the cost trend over time have not been well studied.

Studies show that cancer survivors experiencing financial hardship have decreased medication adherence and lower health-related quality of life (12,13). This decreased adherence likely contributes to the decreased survival seen in cancer patients with financial difficulties related to treatment costs (14). These adverse effects of patient financial hardship are likely extremely pertinent to mRCC patients given the radical change in the treatment landscape for mRCC with the introduction of IO and OAAs (15); however, to our knowledge there has not yet been a study on cost and its impact on adherence among patients with mRCC (16).

To address patient financial toxicities and the increasing burden of treatment costs to our health-care system, it is necessary to understand what is driving the increasing costs of emerging effective but expensive therapies. This is particularly timely, because Medicare part D coverage policy is evolving substantially, with the Inflation Reduction Act lowering the maximum annual out-of-pocket costs to $2000 beginning in 2025. In this study, we used fee-for-service Medicare claims data for patients with mRCC to characterize these treatments and their costs in mRCC over time and their association with medication adherence.

## Methods

### Data source

This study was performed using 100% Medicare claims for fee-for-service beneficiaries who had a diagnosis code for renal cell carcinoma in 2014-2020 from the Chronic Conditions Warehouse (CCW). CCW data contains eligibility, enrollment, medical service use including inpatient (Part A), carrier, and outpatient (Part B), and prescription medication usage (Part D prescription drug event data).

### Study sample

To be included in the study cohort, patients had to have an International Classification of Diseases (ICD)-9 or ICD-10 code of kidney cancer (ICD-9: 198.0; ICD-10: C64*) between 2014 and 2020. Metastatic disease was determined by ICD-9 or ICD-10 codes of metastatic cancer (ICD-9 196.*, 197.*, 198.*; ICD-10 C77.*, C78.*, C79.*) on two claims on separate days less than 1 year apart. The first day became the metastatic index date. Patients were required to be 66 or older at the metastatic index date. To ensure complete data on each patient, we only included patients enrolled in Medicare fee-for-service Parts A, B, and D for 12 months before and after the metastatic index date or until death if it occurred less than 12 months post-index date. We excluded patients with additional malignant tumors, except for nonmelanoma skin cancers. Study selection details were previously reported (17).

For each patient, we examined year of metastatic diagnosis, age at metastatic diagnosis, race, and ethnicity. Socioeconomic variables included dual eligible status (ie, patients also eligible for Medicaid) and Part D low-income cost sharing beneficiaries. Comorbidities were assessed using claims-based Elixhauser Comorbidity Index in the 12 months before metastatic index date (18). Prior year cost (Medicare and patient payments) was also assessed for the 12 months before metastatic index date. We described patient location at diagnosis based on 1) residence in metropolitan area and 2) census geographic region of residence.

### Outcome variables

#### Treatment received

We identified the earliest treatment a person received as OAA, IO, other, or none in the first year after diagnosis. If a patient received both OAA and IO within 60 days of one another, then they were classified in the IO/OAA combination group; patients could not be assigned to more than one category. Using generic names in Part D claims, we identified OAA treatment: sorafenib, sunitinib, pazopanib, everolimus, axitinib, lenvatinib mesylate, cabozantinib s-malate, or tivozanib hydrochloride. We used Healthcare Common Procedure Coding System Codes to identify IO agents: ipilimumab, nivolumab, pembrolizumab, avelumab, interleukin-2, or interferon-alfa.

#### Costs

We summarized Medicare reimbursements and out-of-pocket (OOP) responsibility using claims from outpatient, inpatient, carrier, and pharmacy claims in the 12 months after diagnosis for patients who lived at least 1 month (19,20). We included deductible, copayment, and coinsurance in OOP costs. All costs were adjusted for inflation to 2019 United States dollars (USD) using the consumer price index (CPI) (21). We Windsorized cost outliers to the 99th percentile.

We calculated cost after diagnosis in patients as overall cost and subdivided into components: treatment, inpatient (Intensive Care Unit vs non-Intensive Care Unit), and outpatient (emergency department, pharmacy, office visits, lab work, and other). We then performed sensitivity analysis by examining monthly costs in patients who lived more than or less than 1 year after diagnosis.

#### Adherence

Adherence to OAA therapy was calculated using the Part D prescription drug event claims for patients receiving OAAs as initial therapy. We calculated the percent of eligible days covered from first fill until 90 days (and 180 days in sensitivity analyses) later or death, whichever came earlier.

### Statistical analysis

We examined differences using χ^2^, *t* tests, and Analysis of Variance in our analytic sample by type of initial treatment.

We used generalized linear models with a gamma distribution and log link to assess differences in costs according to treatment categorization (ie, OAA, IO, other). This model produces cost relative ratios and confidence intervals and marginal probabilities approximate per patient Medicare payments and OOP responsibility. Regression analysis included adjustment for patient race and ethnicity, year of diagnosis, age at diagnosis, sex, comorbidity score, socioeconomic factors (living in a metro area, dual eligible for Medicaid and Part D low-income subsidy), receipt of nephrectomy, and prior year Medicare payments.

We assessed the relation between initial 30-day OOP responsibility of OAA categorized as less than $200, $200 to $1000, and greater than $1000 and percent days covered with an OAA agent as continuous and binary “adherent” (>80%, based on prior literature) (22-24) during the first 90 and 180 days after initiation using linear and log-binomial regression, respectively. We focused on OAAs because these are oral medications, with OOP coming from the Part D section of Medicare. We also specifically looked at patients Part D OOP costs exceeding $2000, the new OOP responsibility limit outlined in the Inflation Reduction Act.

Statistical analysis was done using SAS version 9.4 (SAS Institute, Inc, Cary, NC) and Stata version 18.0 (StataCorp, College Station, TX). This study was approved by the Yale Human Investigation Committee.

## Results

### Study sample

We identified 15 407 patients diagnosed with mRCC between 2015 and 2019 and aged 66 or older from Medicare fee-for-service claims. Our sample included predominantly male (61%) and non-Hispanic White (85%) patients. There were 2566 patients (17%) who were dually eligible for Medicaid, and 19% qualified for Part D Low Income Subsidy. Approximately 29% of patients survived less than 6 months, 14% survived 6 to 12 months, and 57% survived more than 12 months; those with survival less than 1 month were excluded from the sample. The majority of patients received treatment in the first year after diagnosis (54%).

### Changes in treatment regimens over time

**Figure 1.**
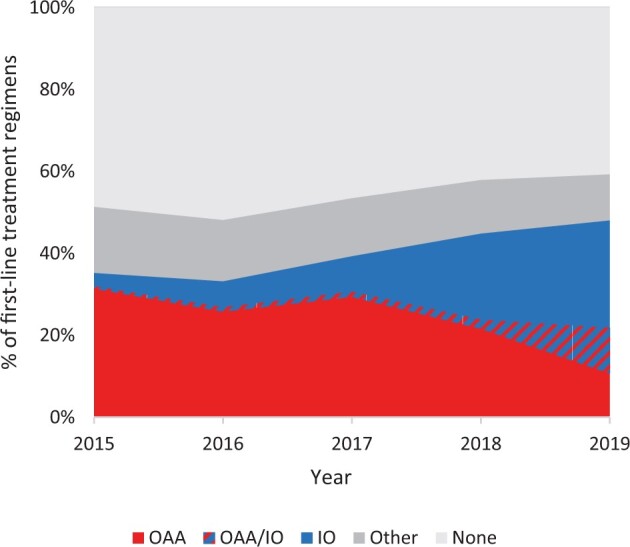
Trends in type of treatment received as first-line therapy over time. We identified the earliest treatment a person received as OAA, IO, other, or none in the first year after diagnosis. If a patient received both OAA and IO within 60 days of one another, then they were classified in the OAA/IO combination group; patients could not be assigned to more than one category. OAA = oral anticancer agent; OAA/IO = oral anticancer agent/immunotherapy combination therapy; IO = immunotherapy.

Treatment types included either OAA alone (3640; 24%), IO alone (2037; 13%), combination IO/OAA (519; 3%), and other (2140; 14%) as their first line (Table 1), with patients assigned to only one category. First-line treatment regimen changed dramatically over the time period studied (Figure 1). OAAs composed 31% of first-line treatment regimens in 2015, compared with 11% in 2019. IO frequency increased from 3% in 2015 to 26% in 2019, and combination IO/OAA increased from 1% in 2015 to 11% in 2019.

**Table 1.** Demographics of study cohort overall and stratified by initial treatment

	Total		Any treatment	OAA	IO	OAA/IO	Other treatment	No treatment	
	N	% (col)	% (row)	% (row)	% (row)	% (row)	% (row)	% (row)	
N	15 407		54%	23.6%	13.2%	3.4%	13.9%	45.9%	Chi-square *P*
**Index year**									
2015	2951	19%	51%	31%	3%	1%	16%	49%	*P* < .001
2016	3073	20%	48%	26%	6%	1%	15%	52%	
2017	3122	20%	53%	29%	9%	1%	14%	47%	
2018	3076	20%	58%	22%	21%	2%	13%	42%	
2019	3185	21%	59%	11%	26%	11%	11%	41%	
**Patient race and ethnicity**									
Non-Hispanic White	12 966	84%	55%	23%	14%	3%	14%	45%	*P* < .001
Non-Hispanic Black	1017	7%	45%	20%	9%	3%	13%	55%	
Asian and/or Pacific Islander	257	2%	54%	26%	12%	5%	11%	46%	
Hispanic	757	5%	56%	31%	10%	3%	12%	44%	
American Indian and/or Alaska Native, Other, Unknown	410	3%	59%	28%	15%	4%	12%	41%	
**Age at diagnosis median (Interquartile range)**	75 (70-80)		74 (70-78)	73 (69-78)	74 (70-79)	73 (70-77)	74 (70-79	76 (71-82)	
66-70	4261	28%	61%	28%	15%	4%	14%	39%	*P* < .001
71-75	4215	27%	59%	25%	14%	4%	15%	41%	
76-80	3307	21%	56%	24%	14%	3%	15%	44%	
81+	3624	24%	39%	16%	10%	2%	12%	61%	
**Patient sex**									
Male	9360	61%	57%	25%	14%	4%	14%	43%	*P* < .001
Female	6047	39%	49%	21%	11%	3%	14%	51%	
**Comorbidity score**									
No conditions	4974	32%	56%	27%	10%	3%	16%	41%	*P* < .001
1-2 conditions	5047	33%	57%	23%	16%	4%	14%	44%	
3+ conditions	5386	35%	50%	21%	14%	3%	12%	52%	
**Frailty index**									
Not frail	10 442	68%	57%	25%	14%	4%	15%	42%	*P* < .001
Likely frail	4965	32%	48%	20%	13%	3%	12%	54%	
**Residence in metropolitan area**									
No	3656	24%	56%	24%	14%	4%	13%	45%	*P* = .006
Yes	11 751	76%	54%	23%	13%	3%	14%	46%	
**Dual eligible**									
No	12 841	83%	56%	23%	15%	4%	14%	44%	*P* < .001
Yes	2566	17%	45%	25%	7%	2%	11%	55%	
**Part D limited income subsidy**									
No	12 448	81%	56%	23%	15%	4%	15%	44%	*P* < .001
Yes	2959	19%	46%	26%	7%	2%	11%	54%	
**Individual limited income subsidy**									
100% premium subsidy, no copay	701	5%	31%	189%	72%	21%	113%	315%	*P* < .001
100% premium subsidy, some copay	2177	14%	51%	54%	37%	9%	32%	100%	
0-25% premium subsidy, some copay	12 529	81%	56%	9%	6%	1%	5%	22%	
**Nephrectomy timing**									
None	11 870	77%	53%	23%	13%	3%	14%	47%	
Pre-Index nephrectomy	2231	14%	58%	26%	15%	4%	13%	42%	
Post-Index nephrectomy	1306	8%	57%	26%	14%	4%	12%	43%	
**Pre-index total cost per 10 000 in 2019 USD**									
Mean (SD)	2.00 (2.58)		1.85 (2.28)	1.97 (2.32)	1.78 (2.26)	1.58 (2.00)	1.80 (2.29)	2.18 (2.89)	
**Survival after index**									
31-60 days	1508	10%	21%	9%	4%	*1%*	7%	79%	
61-90 days	1099	7%	38%	15%	8%	2%	13%	62%	
91-180 days	1877	12%	53%	22%	12%	3%	16%	47%	
181-365 days	2134	14%	71%	28%	19%	4%	21%	29%	
>365 days	8789	57%	58%	26%	14%	4%	13%	42%	

IO = immunotherapy; OAA = oral anticancer agent; OAA/IO = oral anticancer agent/immunotherapy combination therapy; USD = United States Dollars.

### Trends in initial treatment costs

Initial year Medicare payments for mRCC increased by 41% from $60 320 (95% confidence interval [CI] = 58 260 to 62 380) in 2015 to $85 130 (95% CI = 82 630 to 87 630) in 2019 (cost relative ratio [CRR] = 1.41 [95% CI = 1.38 to 1.48]). From 2015 to 2019, costs for patients receiving OAA, IO, or the combination also rose by 41% from $90 700 to $128 200 (CRR = 1.41 [95% CI = 1.35 to 1.48]). In contrast, patients who did not undergo treatment had stable costs around $45 310 (CRR = 0.96 [95% CI = 0.89 to 1.03]).

### Change in treatment costs over time

**Figure 2.**
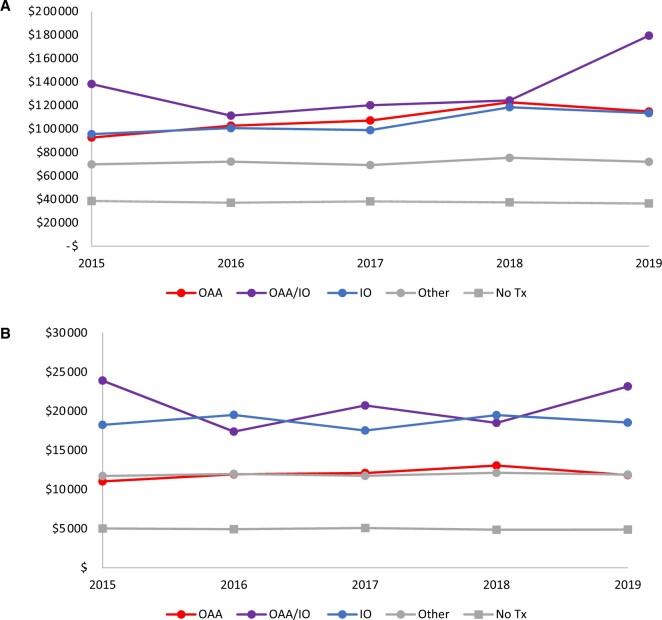
**A)** Mean Medicare payments by treatment type and diagnosis year in 2019 US dollars. **B)** Mean overall out-of-pocket responsibility by treatment type and diagnosis year in 2019 US dollars. OAA = oral anticancer agent; OAA/IO = oral anticancer agent/immunotherapy combination therapy; IO = immunotherapy, Tx = treatment.

In patients who received IO, OAA, or a combination, we divided the Medicare costs into treatment, inpatient, and outpatient-related costs. Treatment-related Medicare payments rose by 79% between 2015 and 2019 (CRR = 1.79 [95% CI = 1.66 to 1.93]), whereas Medicare payments associated with inpatient were stable (Supplementary Table 1, available online). Medicare payments for outpatient care decreased from 2015 to 2019 (CRR = 0.88 [95% CI = 0.81 to 0.95]). Combination therapy had higher treatment and outpatient cost compared with OAA alone (treatment CRR = 1.45 [95% CI = 1.34 to 1.57]; outpatient CRR = 1.13 [95% CI = 1.04 to 0.23]). Medicare payments increased in all treatment groups except patients who did not receive therapy, whereas OOP responsibility remained stable over time for all treatment categories (Figure 2, Supplementary Table 3, A and B, available online).

### Trends in monthly costs

**Figure 3.**
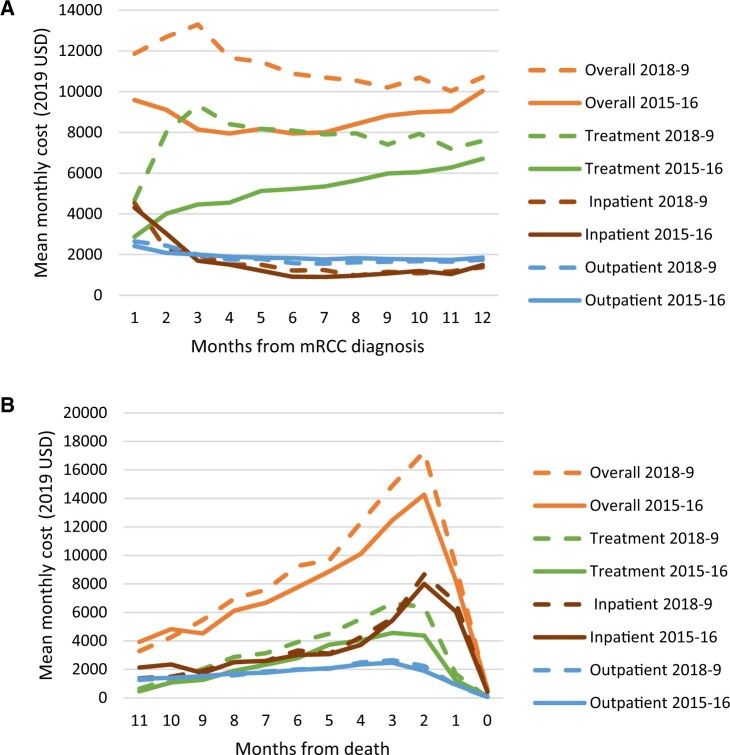
**A)** Trends in monthly Medicare payments by cost category for patients who survived more than 12 months after metastatic renal cell carcinoma (mRCC) diagnosis. Costs are categorized into overall costs, treatment costs, inpatient costs (intensive care vs non–intensive care unit admission), and outpatient (emergency department, pharmacy, office visits, lab work, and other) costs. **B)** Trends in monthly Medicare payments by cost category for patients who lived less than 12 months after metastatic renal cell carcinoma (mRCC) diagnosis. Costs are categorized into overall costs, treatment costs, inpatient costs (intensive care vs non–intensive care unit admission), and outpatient (emergency department, pharmacy, office visits, lab work, and other) costs. USD = United States dollars; OAA = oral anticancer agent; OAA/IO = oral anticancer agent/immunotherapy combination therapy; IO = immunotherapy; Tx = treatment.

In a subgroup who lived longer than a year, cost of care in the first year increased 44% from $69 050 (95% CI = 66 060 to 72 050) in 2015 to $99 510 (95% CI = 95 880 to 103  130) in 2019 (CRR = 1.44 [95% CI = 1.36 to 1.53]). In this subgroup, Medicare payments for inpatient care were higher in the first month after diagnosis and then decreased over the rest of the first year. This pattern was similar when stratified by index year 2015-2016 vs 2018-2019 (Figure 3, A). In the second and third months after diagnosis, treatment costs became the largest component for those diagnosed in 2018-2019. For those who lived less than a year, costs also increased (specifically, inpatient component) in the last 2 months before death (Figure 3, B).

### Combined impact of a rise in treatment costs and a rise in use of more expensive regimens

**Figure 4.**
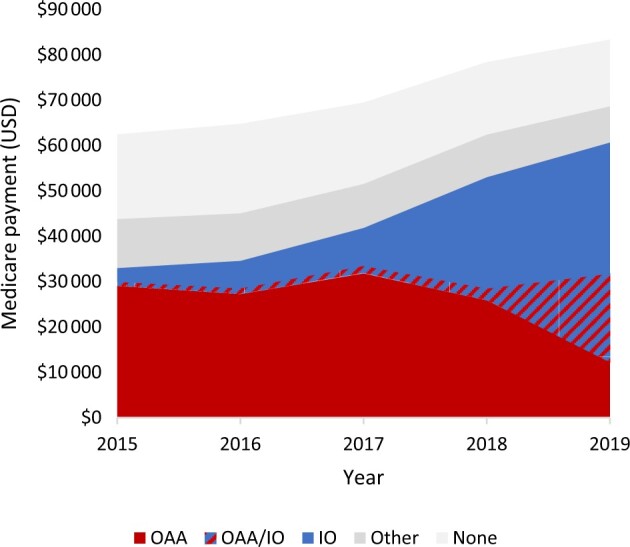
Trends in overall Medicare payments per patient over time by treatment category. We identified the earliest treatment a person received as OAA, IO, other, or none in the first year after diagnosis. If a patient received both OAA and IO within 60 days of one another, then they were classified in the OAA/IO combination group; patients could not be assigned to more than one category. OAA = oral anticancer agent; OAA/IO = oral anticancer agent/immunotherapy combination therapy; IO = immunotherapy.

Taken together, the increases in frequency and mean cost of IO and combination therapies between 2015 and 2019 led to a large increase in average mRCC costs (Figure 4). Medicare payments for patients who received other or no therapy remained stable or decreased slightly over time. The increase of IO and combination therapy led to an increase in Medicare payments for individuals with mRCC, even while the use of OAA steeply decreases.

### Patient perspective: trends in OOP payments and associations with adherence

The OOP responsibility for all patients increased over time from 2015 to 2019 (CRR = 1.48 [95% CI = 1.45 to 1.55]). Treatment-related OOP responsibility in those who had OAA, IO, or combination therapy rose from 2015 to 2019 (CRR = 1.56 [95% CI = 1.40 to 1.73]), whereas inpatient and outpatient OOP responsibility decreased (inpatient CRR = 0.88 [95% CI = 0.81 to 0.96], outpatient CRR = 0.83 [95% CI = 0.76 to 0.89] (Supplementary Table 2, available online). For patients who received other or no treatment, OOP responsibility was similar between 2015 and 2019 (CRR = 0.99 [95% CI = 0.93 to 1.06]).

A large portion (61%) of patients receiving OAAs had total Part D OOP costs greater than $2000; this was less frequent among patients receiving IO monotherapy (18%) but more common in those receiving OAA and IO (73%) (χ^2^*P* < .001). In those who received an IO, OAA, or both, the proportion with OOP more than $2000 decreased over time, with 54% having an OOP responsibility more than $2000 in 2015 compared with 41% in 2019 (χ^2^*P* < .001).

We conducted subgroup analysis of beneficiaries who received OAA as initial treatment. After adjusting for demographic variables, there was a significant association between initial 30-day OOP responsibility for OAA treatment and adherence. Patients with OOP responsibility between $200 and $1000 had 13.8% lower (95% CI = -18.1% to -9.6%) percent days covered in the initial 90 days of treatment compared with those with OOP costs under $200. Beneficiaries with more than $1000 initial 30-day OOP responsibility for OAA had reduced percent days covered (-3.4% [95% CI = -5.6% to -1.2%]) for the 90-day timeframe and compared with patients with less than $200 initial 30-day OOP responsibility. Similar effects were seen for the 180-day timeframe.

## Discussion

Using a national cohort, we found that costs for mRCC among Medicare beneficiaries increased significantly, driven by treatment costs, attributable to both 1) an increase in treatment costs of IO and OAAs and 2) an increase in the proportion of patients receiving IO or combination IO/OAA therapy. Patients who lived more than a year had initial high costs around diagnosis, driven primarily by inpatient costs, followed by stable costs, driven primarily by treatment. Patients who lived less a year experienced rising costs at end of life due to increased inpatient care. Rising OOP costs were negatively associated with adherence (25-28).

The rising cost of new therapies in our data is consistent with the broader trends seen in cancer care. In a prior Surveillance, Epidemiology, and End Results (SEER)-Medicare analysis (29), mRCC costs rose more drastically from 2007 to 2011 than they did during 2002-2006. This was thought to be due to new drugs, which were more expensive on introduction, and their costs accelerated rapidly over time (29). Immunotherapy and targeted agents have increased treatment costs in melanoma (30), non-small cell lung cancer (NSCLC) (31,32), breast cancer, and prostate cancer (33). In contrast, in diseases without IO or OAAs introduced, such as pancreatic (34) and colon (33) cancers, treatment costs have been stable or even decreased over time.

We found that overall costs peaked in the first 3 months after diagnosis, mostly due to inpatient stays and diagnostic workup, and that subsequent costs driven by treatments were lower. Patients who died in the 12 months after diagnosis had a spike in costs about a month before death. This pattern is consistent with the U-shaped cost curve first described by Riley et al. (35): an initial spike around diagnosis, followed by the continuous phase with lower costs, and higher costs at end of life. This trajectory has been demonstrated by several SEER-Medicare analyses in multiple types of cancer (36, 37).

The association of higher OOP costs with lower adherence is well described. In a prior SEER-Medicare analysis (38), we found that higher OOP responsibility and living in an impoverished neighborhood decreased the likelihood of adherence among patients with mRCC. Furthermore, adherence was associated with improved OS (39). Our analysis may underestimate the OOP cost–adherence relationship. Patients without insurance, for example, have been observed to be twice as likely as Medicare patients to report cost affecting their adherence to treatment (38). In breast cancer patients, adherence to adjuvant endocrine therapy has been negatively affected by OOP costs as little as $10 per month in Medicare (40) and $30 per month in commercially insured populations (41). Similar relationships between increasing OOP costs and adherence have also been noted in prostate cancer (42) and NSCLC (43). It is interesting that our findings showed a nonlinear response to OOP costs, with the $200-$1000 OOP costs associated with less adherence compared with the costs above $1000. It is possible that patients with higher incomes may pay more for prescription costs and Part B premiums (44), and the patients who owe more than $1000 are wealthier and better able to afford it than those with a $200-$1000 copay.

The burden of both OOP and Medicare costs are expected to decrease with the implementation of the Inflation Reduction Act (IRA), which caps Part D total OOP costs at $2000 per year, removes the donut hole in which Medicare OOP costs rise suddenly, limits drug price increases to the price of inflation, and allows Medicare to negotiate for specific Part B and Part D drugs (15). This is particularly important to the classes of drugs our paper focuses on, because 41% of patients in 2019 had OOP costs above this threshold.

### Limitations

This study has several limitations. Because this was a Medicare claims only analysis, there were no data on costs for patients with private insurance, Medicare Advantage plans, or uninsured patients. However, this focus on Medicare data is important given the recent passage of the IRA and its anticipated changes. Our study used the 100% Medicare Claims database, which lacks information on patient-level disease severity and cancer-specific survival. Furthermore, by design, we focused on the most recent years of data, which precluded adequate follow-up to assess long-term survival outcomes. Although this is a quantitative study assessing costs, further research is needed to assess patients’ views and experiences of these costs as well as the association of costs with clinical outcomes such as survival. Lastly, it should be noted that various patient factors were associated with treatment receipt, and this in turn could have affected costs. However, the impact of patient factors on direct treatment costs billed to Medicare is thought to be minimal, and further minimized by examining trends over time.

This is one of the first studies examining how the adoption of IO and OAAs affect Medicare and OOP costs, and the first study reporting the overall costs in mRCC. We found that, with the adoption of these new therapies, costs increased significantly, which could have broader implications for Medicare as IO and OAAs are widely adopted. One aim of the IRA is to curb these costs; future studies will have to determine its impact on the ballooning costs of cancer care.

## Supplementary Material

pkae067_Supplementary_Data

## Data Availability

Medicare claims data are available from the Chronic Conditions Warehouse via application through the Research Data Assistance Center (ResDAC) at the University of Minnesota. Research Identifiable Files are available upon application. Per the standard Centers for Medicare & Medicaid Services (CMS) data use agreement, we are not allowed to share any data directly.
