# Supplementary material for: Cost trends of metastatic renal cell carcinoma therapy: the impact of oral anticancer agents and immunotherapy
Source: JNCI Cancer Spectr. 2024 Aug 12;8(5):pkae067. doi: 10.1093/jncics/pkae067 (PMC11376369; doi:10.1093/jncics/pkae067)
Supplement: pkae067_Supplementary_Data [file pkae067_supplementary_data.pdf]

__Supplementary Material__

Supplementary Table 1: Adjusted relative cost ratios in metastatic renal cell carcinoma patients receiving OAA vs IO or IO+OAA combination therapy: Medicare Payments

Abbreviations:
IO: Immunotherapy, OAA: Oral Anti-Cancer Agent, OAA/IO: Oral Anti-Cancer Agent/Immunotherapy combination therapy, CRR: Cost Relative Ratio, CI: Confidence Interval, NHW: Non-Hispanic White, NHB: Non-Hispanic Black, API: Asian or Pacific Islander, OOP: out of pocket

	Overall Costs				**Treatment Costs**				Inpatient Costs				Outpatient Costs			
	CRR	95% CI		p-value	CRR	95% CI		p-value	CRR	95% CI		p-value	CRR	95% CI		p-value
__Treatment Type__																
OAA								*reference*								
IO	0.95	0.92	0.99	0.004	0.75	0.72	0.79	<.001	1.22	1.13	1.31	<.001	1.31	1.25	1.37	<.001
OAA/IO	1.36	1.28	1.43	<.001	1.45	1.34	1.57	<.001	1.11	0.98	1.25	0.089	1.13	1.05	1.23	0.003
__Patient Race and Ethnicity__																
NHW								*reference*								
NHB	1.06	1.00	1.12	0.063	1.07	0.98	1.17	0.119	1.10	0.96	1.26	0.178	0.93	0.84	1.02	0.107
API	1.08	0.97	1.19	0.151	0.93	0.81	1.08	0.351	1.18	0.94	1.49	0.155	1.28	1.10	1.50	0.002
Hispanic	1.14	1.07	1.21	<.001	1.17	1.07	1.28	0.001	1.18	1.03	1.36	0.020	1.01	0.92	1.12	0.762
Other, Unknown	0.99	0.92	1.07	0.873	0.98	0.88	1.09	0.714	1.02	0.85	1.21	0.850	1.05	0.93	1.18	0.410
__Index year__																
2015								*reference*								
2016	1.13	1.08	1.19	<.001	1.26	1.17	1.35	<.001	1.01	0.90	1.13	0.931	1.00	0.93	1.08	0.975
2017	1.17	1.12	1.23	<.001	1.36	1.27	1.46	<.001	0.98	0.87	1.09	0.667	0.96	0.89	1.04	0.319
2018	1.34	1.27	1.41	<.001	1.77	1.65	1.90	<.001	0.97	0.87	1.09	0.623	0.95	0.88	1.03	0.203
2019	1.33	1.26	1.40	<.001	1.79	1.66	1.93	<.001	0.94	0.84	1.06	0.312	0.88	0.81	0.95	0.002
__Index age__																
66-70								*reference*								
71-75	0.96	0.93	1.00	0.030	0.98	0.93	1.02	0.311	0.95	0.88	1.02	0.161	0.95	0.90	1.00	0.068
76-80	0.89	0.86	0.92	<.001	0.88	0.83	0.93	<.001	0.86	0.79	0.94	<.001	0.92	0.87	0.98	0.006
81+	0.74	0.72	0.78	<.001	0.72	0.68	0.76	<.001	0.76	0.69	0.83	<.001	0.79	0.75	0.85	<.001

Female sex	0.93	0.90	0.95	<.001	0.87	0.83	0.90	<.001	1.02	0.96	1.09	0.466	0.98	0.94	1.02	0.290
Elixhauser Comorbidity score																
No conditions									*reference*							
1-2 conditions	0.99	0.95	1.02	0.497	1.00	0.95	1.05	0.873	0.97	0.90	1.06	0.533	0.97	0.92	1.02	0.265
3+ conditions	0.96	0.92	1.00	0.058	0.89	0.84	0.95	<.001	1.09	0.99	1.21	0.080	0.99	0.92	1.06	0.784
Likely Frail	1.00	0.97	1.04	0.984	0.98	0.93	1.03	0.361	1.08	0.99	1.17	0.070	0.99	0.94	1.05	0.788
Metro Residence	1.09	1.06	1.12	<.001	1.01	0.97	1.06	0.542	1.28	1.19	1.37	<.001	1.09	1.04	1.15	<.001
Dual Eligible	0.95	0.86	1.05	0.281	0.93	0.81	1.08	0.340	0.92	0.74	1.16	0.497	1.08	0.93	1.26	0.323
Part D Low Income Subsidy																
0 Premium & 0 Copay									*reference*							
0 Premium & Some copay	1.00	0.91	1.09	0.979	0.97	0.85	1.10	0.590	0.99	0.81	1.22	0.936	1.04	0.91	1.20	0.547
25-100% Premium	0.93	0.82	1.06	0.258	0.94	0.78	1.14	0.544	0.81	0.61	1.09	0.170	1.09	0.89	1.33	0.408
Nephrectomy																
None									*reference*							
Pre-Index	0.97	0.94	1.01	0.154	1.00	0.95	1.06	0.900	1.06	0.97	1.15	0.194	0.84	0.79	0.89	<.001
Post-Index	1.15	1.10	1.20	<.001	0.95	0.89	1.02	0.139	1.67	1.50	1.85	<.001	1.05	0.98	1.13	0.155
Prior Year Medicare Payments + OOP Responsibility per $10,000	1.02	1.02	1.03	<.001	1.00	0.99	1.01	0.467	1.03	1.01	1.04	0.001	1.07	1.06	1.08	<.001

Supplementary Table 2: Adjusted relative cost ratios in metastatic renal cell carcinoma patients receiving OAA vs IO or IO+OAA combination therapy: out of pocket responsibility

Abbreviations:
IO: Immunotherapy, OAA: Oral Anti-Cancer Agent, OAA/IO: Oral Anti-Cancer Agent/Immunotherapy combination therapy, CRR: Cost Relative Ratio, CI: Confidence Interval, NHW: Non-Hispanic White, NHB: Non-Hispanic Black, API: Asian or Pacific Islander, OOP: out of pocket

	Overall Costs				**Treatment Costs**				Inpatient Costs				Outpatient Costs			
	CRR	95% CI		p-value	CRR	95% CI		p-value	CRR	95% CI		p-value	CRR	95% CI		p-value
<u>Treatment Type</u>																
OAA	*Reference*															
IO	1.52	1.46	1.57	<.001	1.88	1.76	2.01	<.001	1.16	1.10	1.23	<.001	1.38	1.32	1.45	<.001
OAA/IO	1.78	1.68	1.88	<.001	2.68	2.41	2.99	<.001	1.10	1.01	1.21	0.032	1.12	1.04	1.22	0.004
<u>Patient Race and Ethnicity</u>																
NHW	*Reference*															
NHB	0.99	0.93	1.06	0.786	0.99	0.87	1.11	0.823	1.09	0.98	1.20	0.114	0.93	0.85	1.02	0.117
API	0.99	0.89	1.10	0.856	0.81	0.66	0.99	0.043	1.00	0.84	1.19	0.978	1.19	1.03	1.39	0.022
Hispanic	1.11	1.04	1.19	0.001	1.16	1.03	1.32	0.016	1.11	1.00	1.23	0.052	1.03	0.94	1.13	0.558
Other, Unknown	1.07	0.99	1.16	0.097	1.18	1.01	1.38	0.038	1.00	0.88	1.14	0.995	1.07	0.96	1.20	0.240
<u>Index year</u>																
2015	*Reference*															
2016	1.11	1.05	1.17	<.001	1.35	1.22	1.49	<.001	0.97	0.89	1.05	0.448	0.99	0.92	1.07	0.843
2017	1.11	1.05	1.17	<.001	1.42	1.28	1.56	<.001	0.97	0.89	1.05	0.458	0.94	0.87	1.01	0.076
2018	1.17	1.11	1.23	<.001	1.62	1.46	1.79	<.001	0.96	0.88	1.04	0.314	0.90	0.84	0.97	0.007
2019	1.12	1.06	1.18	<.001	1.56	1.40	1.73	<.001	0.88	0.81	0.96	0.004	0.83	0.76	0.89	<.001
<u>Index age</u>																
66-70	*Reference*															
71-75	0.99	0.95	1.03	0.563	1.04	0.97	1.11	0.278	0.98	0.93	1.04	0.523	0.95	0.90	1.00	0.040
76-80	0.92	0.88	0.96	<.001	0.91	0.84	0.98	0.009	0.92	0.87	0.98	0.013	0.93	0.88	0.98	0.006
81+	0.80	0.76	0.83	<.001	0.77	0.71	0.83	<.001	0.87	0.81	0.94	<.001	0.80	0.75	0.85	<.001

Female sex	0.95	0.93	0.98	0.002	0.90	0.85	0.96	0.001	1.02	0.97	1.07	0.499	0.98	0.94	1.02	0.383
Elixhauser Comorbidity score																
No conditions									*Reference*							
1-2 conditions	0.99	0.95	1.03	0.494	1.00	0.93	1.08	0.966	0.99	0.93	1.05	0.792	0.97	0.91	1.02	0.215
3+ conditions	0.96	0.92	1.01	0.100	0.93	0.85	1.01	0.093	1.11	1.03	1.19	0.007	0.96	0.90	1.03	0.236
Likely Frail	0.99	0.95	1.03	0.573	1.00	0.93	1.07	0.937	1.04	0.98	1.11	0.175	0.99	0.94	1.05	0.741
Metro Residence	1.00	0.97	1.03	0.953	1.03	0.97	1.09	0.405	1.10	1.04	1.15	0.001	0.95	0.90	0.99	0.020
Dual Eligible	0.97	0.87	1.08	0.549	0.87	0.71	1.07	0.190	0.91	0.77	1.08	0.279	1.10	0.95	1.28	0.218
Part D Low Income Subsidy																
0 Premium & 0 Copay										*Reference*						
0 Premium & Some copay	1.04	0.94	1.14	0.435	1.19	0.99	1.42	0.066	0.97	0.83	1.13	0.671	1.07	0.94	1.23	0.308
25-100% Premium	1.44	1.25	1.66	<.001	2.54	1.95	3.30	<.001	0.79	0.63	0.98	0.033	1.36	1.12	1.65	0.002
Nephrectomy																
None									*Reference*							
Pre-Index	0.97	0.93	1.01	0.104	1.02	0.94	1.10	0.659	1.09	1.03	1.16	0.006	0.86	0.82	0.91	<.001
Post-Index	1.08	1.03	1.13	0.003	0.92	0.84	1.01	0.095	1.56	1.44	1.69	<.001	1.05	0.98	1.13	0.135
Prior Year Medicare Payments + OOP Responsibility per $10,000	1.02	1.01	1.03	<.001	0.99	0.97	1.00	0.047	1.00	0.99	1.01	0.555	1.05	1.04	1.07	<.001

Supplementary Table 3a: Mean Medicare Payments by Treatment Type and Diagnosis Year in 2019 U.S. Dollars

Abbreviations: IO: Immunotherapy, OAA: Oral Anti-Cancer Agent, OAA/IO: Oral Anti-Cancer Agent/Immunotherapy combination therapy

	OAA	IO	OAA/IO	Other	None
2015	$92600<br>(95% CI 87680-97510)	$95410<br>(95% CI 80430-110390)	$138100<br>(95% CI 89900-186310)	$69660<br>(95% CI 64630-74690)	$38480<br>(95% CI 36820-40150)
2016	$102650<br>(95% CI 97060-108230)	$100540<br>(95% CI 89540-111540)	$111180<br>(95% CI 82600-139770)	$71970<br>(95% CI 66850-77080)	$36970<br>(95% CI 35540-38390)
2017	$107000<br>(95% CI 101580-112410)	$98820<br>(95% CI 89610-108030)	$120020<br>(95% CI 91850-148190)	$69060<br>(95% CI 64040-74070)	$38170<br>(95% CI 36620-39710)
2018	$122540<br>(95% CI 115260-129820)	$118320<br>(95% CI 111180-125450)	$124120<br>(95% CI 101550-146700)	$75270<br>(95% CI 69520-81020)	$37310<br>(95% CI 35710-38920)
2019	$114680<br>(95% CI 105220-124140)	$113350<br>(95% CI 107320-119370)	$179320<br>(95% CI 164740-193900)	$71870<br>(95% CI 66080-77670)	$36320<br>(95% CI 34760-37890)

Supplementary Table 3b: Mean Out-of-Pocket (OOP) Responsibility by Treatment Type and Diagnosis Year in 2019 U.S. Dollars

Abbreviations: IO: Immunotherapy, OAA: Oral Anti-Cancer Agent, OAA/IO: Oral Anti-Cancer Agent/Immunotherapy combination therapy

	OAA	IO	OAA/IO	Other	None
2015	$11020 (95% CI 10520-11520)	$18240 (95% CI 15800-20680)	$23900 (95% CI 16800-31000)	$11710 (95% CI 10990-12430)	$5010 (95% CI 4820-5190)
2016	$11920 (95% CI 11370-12470)	$19510 (95% CI 17700-21330)	$17380 (95% CI 13580-21180)	$11970 (95% CI 11250-12700)	$4920 (95% CI 4760-5080)
2017	$12080 (95% CI 11560-12600)	$17520 (95% CI 16130-18910)	$20720 (95% CI 16590-24860)	$117300 (95% CI 11010-12450)	$5070 (95% CI 4890-5240)
2018	$13040 (95% CI 12380-13700)	$19480 (95% CI 18490-20480)	$18490 (95% CI 15630-21360)	$12110 (95% CI 11330-12900)	$4850 (95% CI 4670-5030)
2019	$11830 (95% CI 11000-12660)	$18540 (95% CI 17700-19380)	$23150 (95% CI 21550-24750)	$11890 (95% CI 11070-12710)	$4860 (95% CI 4680-5040)
